# Access to Care Among Persons With End Stage Renal Disease After Enrollment in Medicare Advantage

**DOI:** 10.1093/geroni/igaf122.3522

**Published:** 2025-12-31

**Authors:** Denise Tyler, Emily Gadbois, Joan Brazier, Amal Trivedi

**Affiliations:** Miami University, Hancock, Maine, United States; Brown University School of Public Health, Providence, Rhode Island, United States; Brown University, Providence, Rhode Island, United States; Brown University, Providence, Rhode Island, United States

## Abstract

Prior to the 21st Century Cures Act, enrollment in Medicare Advantage (MA) by people with end-stage renal disease (ESRD) was only allowed in limited circumstances. Enrollment in MA by those with ESRD has grown by over 70 percent since the Cures Act, but little is known about the effects of this policy change on those with ESRD and the organizations involved, such as MA plans and dialysis providers. This study, part of a larger mixed methods study on this topic, used qualitative methods to explore access to health care and other services by those with ESRD after enrollment in MA. We conducted 48 interviews with representatives from MA plans, dialysis organizations, and kidney care management companies and qualitatively analyzed these to identify key themes. Participants reported that MA enrollment has impacted patient access to dialysis, health care providers, transplant services, and supplementary benefits. Participants reported that the use of home dialysis modalities had possibly increased, that patients sometimes neglected to consider reduced access to other providers, especially specialists, when selecting MA, and that network restrictions on transplant centers may be affecting patients’ ability to receive transplants. Finally, while some touted the benefits of vision and dental coverage for this population, others reported limited coverage and networks resulting in patients being unable to utilize these benefits. These results suggest that older adults with ESRD may need additional information and resources to ensure their insurance decisions don’t affect their access to needed health care, services, and preferred providers.

